# Advances in Pharmacotherapy of Hepatocellular Carcinoma: A State-of-the-Art Review

**DOI:** 10.1159/000520095

**Published:** 2021-10-13

**Authors:** Florian P. Reiter, Najib Ben Khaled, Liangtao Ye, Changhua Zhang, Max Seidensticker, Mark op den Winkel, Gerald Denk, Andreas Geier, Enrico N. De Toni

**Affiliations:** ^a^Division of Hepatology, Department of Medicine II, University Hospital Würzburg, Würzburg, Germany; ^b^Department of Medicine II, University Hospital Munich, Munich, Germany; ^c^German Cancer Consortium (DKTK), Partner Site Munich, Munich, Germany; ^d^Center for Digestive Diseases, The Seventh Affiliated Hospital, Sun Yat-sen University, Shenzhen, China; ^e^Department of Radiology, University Hospital Munich, Munich, Germany

**Keywords:** Hepatocellular carcinoma, Pharmacotherapy, Immunotherapy

## Abstract

**Background:**

Due to the number of emerging new treatment options, the systemic treatment of hepatocellular carcinoma (HCC) is rapidly changing. We provide here an overview of the current landscape of systemic treatment of HCC and discuss its potential future development.

**Summary:**

HCC is a leading cause of tumor-related death worldwide. Despite the efforts aimed at reducing the prevalence of HCC through vaccination and antiviral treatment, and the implementation of screening programs for early tumor detection, most patients are diagnosed with or progress to advanced HCC. For approximately 10 years, sorafenib has been the only effective systemic treatment available for these patients. Recently, however, a number of new systemic compounds, comprising several multi-kinase inhibitors and immune-checkpoint inhibitors, have been approved for treatment of HCC. These new agents are opening a plethora of therapeutic options for the future therapy of HCC.

**Key Messages:**

The rapid progress in the treatment of HCC raises the question of the optimal combination and sequence of these agents in the treatment of patients with advanced disease. The substantial improvements in terms of objective response and survival indicate that the use of immune-checkpoint inhibitors-based treatment combinations may be extended to patients with intermediate-stage HCC.

## Introduction

Hepatocellular carcinoma (HCC) is a leading cause of cancer-related mortality worldwide [1]. Despite the implementation of screening programs for early detection of HCC [2, 3], the majority of patients are diagnosed at a stage not amenable to curative treatment (Barcelona-Clinic Liver Cancer Classification [BCLC-B] or higher according to the BCLC) [4]. Furthermore, recurrence and/or progression to an advanced stage are frequent, even when HCC is treated in curative intention [4]. Another challenge in the treatment of HCC is represented by the fact that most patients affected by this tumor suffer from an underlying liver disease, which per se accounts for a poor prognosis and represents a significant limitation for the treatment of HCC.

Fortunately, the landscape of HCC treatment has rapidly evolved in recent years, especially with the development of regimens based on tyrosine-kinase inhibitors (TKIs) or vascular endothelial growth factor (VEGF) inhibitors and, more recently, immunotherapy [5, 6, 7, 8, 9]. This positive development brings new hopes, along with new challenges which need to be addressed in the future as follows: which is the optimal combination of agents and sequence of treatment in patients with advanced HCC? Can response to immune-checkpoint inhibitors be predicted by biomarkers? Is the time ripe to use systemic treatment in the therapy of earlier stage HCC? We provide an overview of the agents for systemic treatment of HCC currently approved or undergoing clinical investigation and discuss the future perspectives of HCC treatment.

## Materials and Methods

**Fig. 1:**
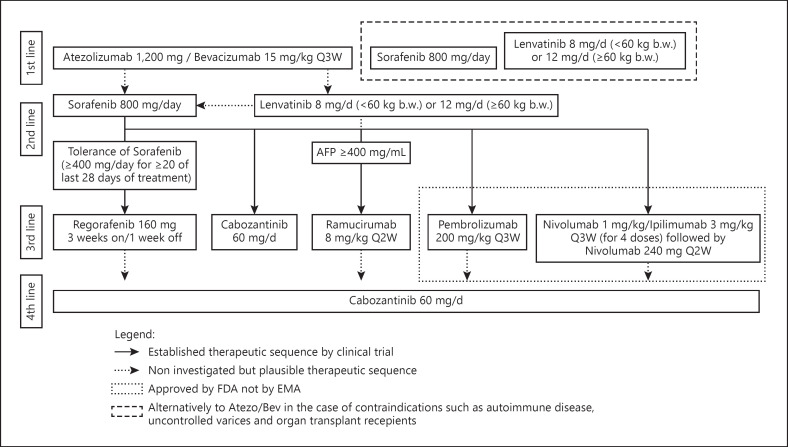
Sequential treatment of HCC. The figure illustrates the available therapies of HCC and their sequential use. The noninterrupted arrow-lines indicate therapies that were investigated in the illustrated sequence by clinical trials. The interrupted therapies exemplify plausible therapeutic sequences that were not or not accurately investigated in the present form. The box with the dotted lines shows therapies that are approved by the FDA but not by the EMA. Of note is that patients with autoimmune disease, uncontrolled varices, and organ transplant recipients might not be optimal candidates for a first-line therapy with atezo/bev (box with interrupted lines). References: *IMbrave150* [9]*; SHARP* [18]*; REFLECT* [7]*; RESORCE* [5]*; CELESTIAL* [6]*; REACH II* [8]*; CheckMate 040* [29]*; Keynote 224* [31]*and 240* [32]*; CheckMate 040* [33]. HCC, hepatocellular carcinoma; Q2W, every 2 weeks; Q3W, every 3 weeks; EMA, European Medicines Agency.

This review is based on a MEDLINE search for articles on systemic treatment of HCC published between 2010 and September 2021, including reports on clinical trials, official guidelines, and expert opinion articles. This review also includes opinions of the authors emerging from the clinical practice and the experience in the interdisciplinary treatment of HCC. Figure 1 was created using GraphPad Prism (CA, USA).

### Current Indications for Systemic Therapy of HCC

Systemic treatment is currently recommended for patients with advanced-stage HCC (as defined by the presence of macroscopic vascular invasion or extrahepatic spread) or for patients who do not qualify for surgery, locoregional treatment, or transplantation, owing to the localization, number, and/or the size of lesions [10]. Poor liver function represents an important factor limiting the treatment of HCC in all tumor stages. In patients with impaired liver function (defined by the presence of Child-Pugh B) not eligible for liver transplantation, tumor-specific treatment provides less benefits compared to patients with preserved liver function [10]. Liver function is one of the most important determinants of survival in these patients. Thereby, treatment of HCC in patients with impaired liver function might have a disadvantageous effect on survival due to potential treatment-related deterioration of liver function [11, 12]. It has been proposed that sorafenib may be safely used in patients with Child-Pugh B. However, randomized data on the actual benefit of treatment are lacking [13, 14, 15]. The role of liver function in determining the survival of HCC patients is exemplified by the real-world data showing a shorter median overall survival (mOS) in patients with Child-Pugh B [16]. In the following paragraphs, we provide a review of the most relevant clinical studies on the treatment of advanced HCC, including the results of early phase trials on promising, but yet not approved compounds and of their combinations.

## First-Line Therapies

### Atezolizumab and Bevacizumab

The IMbrave150 trial (NCT03434379) assessed the combination of atezolizumab (anti-PD-L1) and bevacizumab (VEGF inhibitor) versus sorafenib in the treatment of patients with advanced HCC. This trial demonstrated clear superiority of atezolizumab/bevacizumab (atezo/bev) over sorafenib in all relevant endpoints (mOS, progression-free survival [PFS], objective response rate (ORR), and quality of life (Table 1) [9]). In particular, the ORR was remarkably higher in the atezo/bev arm than in the sorafenib arm according to both Response Evaluation Criteria in Solid Tumors (RECIST) version 1.1 and to modified RECIST (mRECIST) (35.4% vs. 13.9% according to mRECIST) (Table 1) [17]. Up to 12% of all patients achieved a complete remission (CR) with the investigational combination, while CRs with sorafenib were observed in 2.5% of cases. These effects of immunotherapy did not only translate into a survival benefit with an mOS of 19.2 months versus 13.4 months in the sorafenib arm [17] but also the combination of atezo/bev was also superior to sorafenib in preventing the deterioration of quality of life [9]. Although serious adverse events (AEs) were observed more frequently in patients treated with atezo/bev (38% vs. 30.8%), no specific events could be identified being responsible for this increased incidence [9]. The 3 most common AEs in the investigational combination arm were hypertension (29.8%), fatigue (20.4%), and proteinuria (20.1%). Two of which (hypertension and proteinuria) are commonly observed AEs of bevacizumab. Due to the efficacy, acceptable safety and tolerability of atezo/bev, this regimen has become the current standard of care for first-line systemic treatment of HCC. However, some patients' categories were excluded from the trial, including patients with a history of autoimmune disease, patients with Child-Pugh B or higher, patients with untreated or incompletely treated esophageal or gastric varices, and organ transplant recipients [9]. Therefore, further studies are needed to assess the effect of atezo/bev in these patients' subgroups.

### Sorafenib

Sorafenib is a TKI with high affinity for Raf-1, B-Raf, VEGF receptors 1–3, and platelet-derived growth factor (PDGF) receptor β. Sorafenib was approved in 2007, following positive results from the SHARP (NCT00105443) [18] and the Asia-Pacific trial (NCT00492752) [19] (mOS was reported 10.7 vs. 7.9 months in the SHARP trial and 6.5 vs. 4.2 in the Asia-Pacific trial [18, 19] [Table 1]). Sorafenib represented a milestone in the treatment of HCC as the first effective systemic treatment for a tumor traditionally regarded as untreatable. For approximately 10 years, until the approval of regorafenib [5], sorafenib remained the only established systemic therapy for advanced HCC. Sorafenib showed constant efficacy and tolerability in clinical practice (illustrated by its best mOS of 13.4 months, ORR of up to 13.9%, partial response [PR] rates of up to 11.4%, and CR of up to 2.5% according to mRECIST in the IMbrave150 study when used as a comparator [17]). The most common AEs were diarrhea, palmar-plantar erythrodysesthesia, and fatigue which were reported to occur in 39%, 21%, and 22% of patients, respectively [18]. Treatment-related adverse reactions often result in poor tolerability and represent a frequent cause for treatment discontinuation in approximately 20% [20]. Nevertheless, occurrence of treatment-related adverse reactions and in particular skin reactions may indicate a better survival [20]. In our opinion, sorafenib still reflects a valuable effective option for treatment of advanced HCC, especially for patients who are not amendable to immunotherapy-based regimens and/or are not tolerating treatment with lenvatinib.

### Lenvatinib

Lenvatinib is a TKI with high potency against VEGF receptors 1–3, fibroblast growth factor (FGF) receptors 1–4, PDGF receptor α, RET, and KIT. Lenvatinib was approved after the REFLECT trial (NCT01761266) [7], a study evaluating lenvatinib versus sorafenib in the first-line treatment of HCC that met its predefined endpoint of noninferiority in regard to mOS. PFS and the ORR were significantly higher in the lenvatinib arm (Table 1). The most common AEs in the lenvatinib group were hypertension (42%), diarrhea (39%), decreased appetite (34%), and weight loss (31%), while palmar-plantar erythrodysesthesia was less frequent in the sorafenib arm (27% vs. 52%) [7]. Lenvatinib is currently approved as a possible alternative to sorafenib for treatment-naive patients with advanced HCC (Table 1; Fig. 1). The choice whether to use lenvatinib or sorafenib as the first-line treatment option is generally guided by the different spectra of AEs of these agents. However, some experts are of the opinion that the higher response rates observed with lenvatinib may justify the use of this agent in situations where downsizing of tumor volume may represent a therapeutic goal [7].

### Promising Upcoming First-Line Study (Pembrolizumab and Lenvatinib [Not Yet Approved])

The combination of pembrolizumab and lenvatinib was assessed by the open-label phase Ib trial Keynote-524 (NCT03006926) [21]. In contrast to the abovementioned trials, the reported results from this study lack a comparator. In this study, an ORR of 46% (mRECIST) and an mOS of 22 months suggest meaningful efficacy. The most common AEs were hypertension (36%), diarrhea (35%), and fatigue (30%) [21]. Based on these first encouraging results, combined pembrolizumab and lenvatinib is being investigated by a phase III trial as first-line treatment for advanced HCC (LEAP-002; NCT03713593).

### Other First-Line Studies

The CheckMate 459 study (NCT02576509) investigated the efficacy of nivolumab (anti-PD-1) compared with sorafenib. Although this study did not reach its primary endpoint, meaningful response rates showing an ORR of 15% and a numerical benefit in mOS of 16.4 months (vs. 14.7 months with sorafenib) were reported [22]. Given the current development, it is possible that nivolumab will find an area of application in the treatment of HCC, probably in combination with other drugs and/or interventions. The results of the Himalaya trial (NCT03298451) investigated the combination of tremelimumab (anti-CTLA-4) and durvalumab (anti-PD-L1) versus sorafenib in the first-line setting [23]. According to a recent press release the combination met its primary end point of OS superiority compared to sorafenib. Here we are waiting for the publication of these results from a scientific organ.

As a further first-line phase III trial the COSMIC-312 (NCT03755791) investigated the combination of atezo/cabozantinib versus sorafenib. Here, the investigating company recently released that the interim analysis demonstrated that one of its primary endpoints (prolonging PFS) was reached in the combination arm compared to the standard arm, while OS showed only a trend at this early point of analysis.

Donafenib is a new version of sorafenib that includes deuterium. By this modification, the stability and bioavailability of the molecule are increased [24]. Furthermore, this leads to a reduction of metabolites in the gastrointestinal tract and may thereby reduce AEs [24]. The compound was investigated as first-line treatment in comparison with sorafenib in a Chinese population of patients with Child-Pugh A or B (≤7 Pts.) liver function (NCT02645981) [25]. HBV was reported as etiological factor in 90% of patients included in the study. This positive trial met its endpoint of increasing survival versus sorafenib (mOS were, respectively, 12.0 and 10.1 months) although ORR, disease control rate, and PFS did not differ between the 2 treatment arms [25]. Common AEs related to donafenib arm were palmar-plantar erythrodysesthesia (50%), increase in aspartate aminotransferase (AST) (23%), increase in blood bilirubin (19%), decrease in platelet count (28%), and diarrhea (30%).

Donafenib is currently not approved in Europe. The positive results of this trial warrant for further investigation of this agent in a broader population of patients from different geographic areas and with different etiological agents.

Sintilimab was recently assessed in combination with a bevacizumab biosimilar (IBI305) versus sorafenib by a phase II-III trial conducted in China (ORIENT-32; NCT03794440) [26]. The combination of sintilimab and IBI305 demonstrated a significantly higher mOS and PFS than sorafenib (mOS not reached vs. 10.4 months; IRRC-assessed median PFS 4.6 months vs. 2.8 months). Common grade 3–4 AEs were hypertension (14%) in the sintilimab and IBI305 and palmar-plantar erythrodysesthesia (12%) in the sorafenib group [26]. Since this trial was exclusively conducted in China and in patients with chronic HBV, the results of this study will have to be confirmed in a wider cohort of patients.

### “After First-Line” Therapies

In this chapter, we provide an overview of second-line therapies for the treatment of HCC. The majority of the mentioned trials investigated the efficacy of the study drugs after sorafenib. This is of importance as the studies do not formally report the efficacy of these agents after alternative first-line treatments such as atezo/bev and lenvatinib. Future studies need to account for the fact that new first-line treatments for HCC are available, in order to evaluate the optimal sequence of therapy.

### Regorafenib

Regorafenib is a TKI structurally related to sorafenib with a distinct spectrum of kinase inhibition and pharmacological activity [5]. In the RESORCE trial (NCT01774344), patients with preserved liver function (Child-Pugh A) who tolerated sorafenib but experienced a radiological disease progression during treatment with this agent, were randomized to receive regorafenib or placebo. Regorafenib was administered in a 4-week cycle with 3 weeks on and 1 week off regorafenib. The trial showed an improved survival in the experimental arm [5] (mOS of 10.6 months compared to 7.8 with placebo) (Table 2). The most common reported AEs reported were palmar-plantar erythrodysesthesia (53%), diarrhea (41%), and fatigue (40%).

A survival follow-up of patients treated with regorafenib in the context of the RESORCE trial reported a mOS of 26 months for regorafenib-treated patients [27]. Thereby, regorafenib represents today a valuable option in second-line setting for patients who tolerated sorafenib.

### Cabozantinib

Cabozantinib, an inhibitor of VEGF receptors 1–3, MET and AXL, was investigated by the phase III CELESTIAL trial (NCT01908426) [6]. This study reached its endpoint of improving mOS versus placebo (10.2 vs. 8 months) with placebo. Furthermore, PFS was also improved to 5.2 months compared to 1.9 months (Table 2). Cabozantinib was approved for treatment of patients who had received a prior sorafenib treatment. Toxicity was generally manageable, with palmar-plantar erythrodysesthesia (46%), hypertension (29%), increased AST levels (22%), fatigue (45%), and diarrhea (54%) being reported as the most common AEs.

Patients who had received up to 2 lines of systemic treatments were allowed to participate to the trial which thus included of patients who had received regorafenib, lenvatinib, ramucirumab, and anti-PD-1/PD-L1 directed therapies. Although the patients' population which had received more than one line of treatment represented a small minority of the overall study population, cabozantinib is generally regarded as a potential option for patients who received more than one line of previous systemic treatment (Fig. 1).

### Ramucirumab

Ramucirumab is an IgG1 monoclonal antibody directed against VEGF-2 that was investigated in patients pretreated with sorafenib and demonstrated an improvement of mOS in patients with α-fetoprotein (AFP) levels of 400 ng/mL or higher (REACH-II) (NCT02435433), effects that did not reach significance in the overall population (REACH) (NCT01140347) [8, 28]. Grade 3 or worse treatment-emergent AEs were hypertension (13%), hyponatremia (6%), and increased AST (3%). In light of these results, ramucirumab gained approval for treatment of HCC in the second-line for patients with an AFP level of 400 ng/mL or greater.

### Nivolumab

The CheckMate 040 trial (NCT01658878) is a phase 1/2 dose escalation and expansion trial evaluating the use of nivolumab (anti-PD-1) in several different treatment arms [29]. After the initial dose-finding phase, 214 patients (57 patients were reported as progressors on sorafenib) were included in the dose expansion phase. In this collective, meaningful effects in terms of mOS of (13.2 months) and the ORR (21%) were observed (Table 3). The most frequent AEs reported included rash (23%), pruritus (19%), decreased appetite (10%), and diarrhea (10%).

Due to these encouraging results, nivolumab was granted approval by the Food and Drug Administration (FDA) for patients previously treated by sorafenib, thus becoming the first approved immunotherapeutic agent for the treatment of HCC. A subsequent phase III trial (CheckMate 459, NCT02576509) conducted to investigate the efficacy of nivolumab versus sorafenib in the survival of treatment-naive patients with advanced HCC failed to reach its primary endpoint. Meaningful response rates (15% ORR) and mOS (16.4 vs. 14.7 months in the sorafenib arm) were reported [22]. Nonetheless, approval for nivolumab monotherapy in the second-line setting was recently withdrawn by the FDA [30]. Due to its promising biological activity, the clinical assessment of nivolumab is being continued in combination with several agents.

### Pembrolizumab

Like nivolumab, pembrolizumab (anti-PD-1) demonstrated promising signs of activity in a phase II trial (Keynote-224, NCT02702414 [31]) in patients with HCC previously treated with sorafenib and was approved by the FDA (although not by the European Medicines Agency). Unfortunately, the subsequent phase III trial (Keynote-240 [NCT02702401] failed to reach pre-specified statistical significance in its co-primary endpoints mOS and PFS despite unequivocal signs of efficacy (ORR was 18.3% accordingly to RECIST 1.1) [32] (Table 3). Pembrolizumab, like nivolumab, is being assessed in trial of combined treatment with other agents.

### Nivolumab and Ipilimumab

Nivolumab was investigated in combination with ipilimumab (anti-CTLA-4) in one of the arms of the CheckMate 040 trial (NCT01658878) [33]. Patients were randomized 1:1:1 to receive nivolumab 1 mg/kg plus ipilimumab 3 mg/kg every 3 weeks for 4 doses followed by nivolumab 240 mg intravenously every 2 weeks (arm A, “Ipi^high^”); nivolumab 3 mg/kg plus ipilimumab 1 mg/kg every 3 weeks for 4 doses followed by nivolumab 240 mg intravenously every 2 weeks (arm B); and nivolumab 3 mg/kg every 2 weeks plus ipilimumab 1 mg/kg every 6 weeks (arm C). In this trial, 146 of 148 patients (99%) had received prior treatment with sorafenib. The most promising results were reported for arm A (“Ipi^high^”) with an mOS of 22.8 months, an ORR of 32%, and CR in 8% of patients.

In arm A (“Ipi^high^”), any-grade treatment-related AEs leading to discontinuation of either drug occurred in 18% of cases. AEs reported most frequently for arm A (“Ipi^high^”) were pruritus (45%), rash (29%), diarrhea (24%), and AST increase (20%). Results from this subgroup of 50 patients (arm A [“Ipi^high^”]) included in this arm led to approval by the FDA.

However, the combination is as of today not approved by the European Medicines Agency. These results, which need confirmation in wider patients' cohorts, indicate a higher effectiveness of this combination at higher doses of ipilimumab.

### Apatinib

Apatinib is a VEGF inhibitor investigated in second-line treatment in patients who were previously refractory or intolerant to at least one line of systemic chemotherapy or targeted therapy. This trial (AHELP; NCT02329860) was exclusively performed in China [34] and demonstrated an improved mOS in the apatinib arm versus the placebo (8.7 months vs. 6.8 months) and an acceptable safety profile. The most common treatment-related grade 3 or 4 AEs were hypertension, palmar-plantar erythrodysesthesia, and decreased platelet count, which were observed in 28%, 18%, and 13% of patients, respectively.

### Adjuvant Therapeutic Concepts

Sorafenib failed to demonstrate effects in the adjuvant setting after resection or ablation (STORM trial; NCT00692770) [35]. Due to the high recurrence rates after surgical resection or ablation treatment, the lack of effective regimens of adjuvant treatment remains an important unmet medical need. Currently, several adjuvant CPI-based regimens (e.g., Keynote-937: pembrolizumab vs. placebo; NCT03867084, CheckMate 9DX: nivolumab vs. placebo; NCT03383458) or CPI-based treatment combinations (e.g., IMbrave050: atezo/bev vs. placebo; NCT04102098, Emerald 2: durvalumab vs. durvalumab/bev vs. placebo; NCT03847428) are being investigated in clinical trials of adjuvant treatment [4].

### Choice of Treatment

The combination of atezo/bev is currently the established first-line treatment option for patients with advanced disease (Fig. 1). However, immunotherapy might not be the first choice for all patients, and as the use of atezo/bev is being extended from the well-defined setting of clinical trials to the daily practice, special attention should be paid in the treatment of patients who would not fulfill the stringent inclusion criteria of the trials which led to the approval of these agents. The possibility of the occurrence of autoimmune reactions, of hypertensive crises, or of bleeding needs to be carefully considered in patients with predisposing factors.

However, the presence of an autoimmune disease in the patient's medical history does not always represent an absolute contraindication to the use of immunotherapy and the severity of the underlying condition and the affected organ (e.g., psoriasis vs. autoimmune hepatitis), the possibility of monitoring for an exacerbation of the underlying autoimmune disease (e.g., as allowed by serial assessment of transaminases vs. repeated lung function tests), and most importantly, the potential oncological benefit must be taken into account. Immune treatment cannot be recommended as first-line therapy in organ transplant recipients although several reports show that organ rejection does not invariably occur in patients treated with immune-checkpoint inhibitors [36]. The occurrences of potentially life-threatening bleeding or hypertensive crisis are 2 potential consequences attributed to the effect of bevacizumab. Bleeding risk is of particular importance in patients with HCC who, due to the underlying liver disease, are at increased risk of esophagus varices, impairment of coagulation, and thrombocytopenia, conditions which are predisposing for a potentially life-threatening outcome. Routine endoscopic examination to treat esophageal varices should thus be recommended in every patient with HCC prior to treatment initiation. Several promising regimens of immunotherapy-based treatment are being currently investigated and are likely to be approved in the near future; the expected wider variety of therapeutic options may allow to guide the choice of one combination according to the likelihood of occurrence of an AE in individual patient situations. Bevacizumab-free regimens, for example, based on the administration of 2 combined immunotherapeutics (such as the combination of nivolumab/ipilimumab or durvalumab/tremelimumab discussed above), might represent a future alternative for patients at risk of bleeding or with poorly controlled arterial hypertension. Patients' informed consent after a thorough discussion of the individual risks and benefits of the regimens available remain in any case the mainstay of treatment choice.

For patients deemed not eligible for immunotherapy, TKI inhibitors may represent an option for initial treatment. The development of TKI-based sequential treatment substantially increased patients' survival, and long-term disease control can be achieved with these agents [37]. It is generally accepted that for patients who experience a radiological progression on atezo/bev, TKI treatment should be initiated (Fig. 1). The choice of treatment however should consider several factors, including the specific efficacy, the spectrum of adverse reaction, and the label of approval in individual contexts. For instance, the choice between lenvatinib and sorafenib as the first-line treatment option is generally guided by different spectra of AEs of these agents (hypertension being more often reported for lenvatinib 42% vs. 30% [7]). However, for some experts, the higher response rates observed with lenvatinib may justify the use of this agent preferentially in situations where downsizing of tumor volume may represent an urgent therapeutic goal. Finally, the choice of the first TKI will have to consider the approval label. In several countries, lenvatinib is approved for therapy of treatment-naive patients and the choice of using sorafenib first might preclude the use of lenvatinib in the subsequent lines of treatment.

## Perspective and Future Challenges

### Need for Biomarkers of Response

Systemic therapy for HCC is undergoing a rapid evolution due to the availability of several effective agents used in different lines of treatment. A peculiarity of HCC is represented by the fact that most patients suffer from concurrent liver disease and that tumor progression invariably causes a potentially fatal worsening of liver function. Therefore, identifying biomarkers capable of predicting which of the available agents will be most effective in individual patients at the earliest time point remains a priority in HCC. Unfortunately, despite of major efforts, no reliable biomarker of response capable of predicting the effect of sorafenib could be established. The only validated biomarker of response so far available is represented by AFP, which determines with a cutoff of 400 ng/mL the indication to treatment with ramucirumab [8, 28]. Although reliable biomarkers of response to immune treatment for HCC are not available yet [38], the expression of PD-L1 is a validated response predictor to the action of immunotherapy in some entities (such as lung or bladder cancer [39]) and indications that PD-L1 expression plays a role in predicting response in HCC deserve further investigation [29, 31, 38]. Pembrolizumab gained tumor-agnostic approval for microsatellite instability-high (MSI-H) tumors. However, an MSI-H status is rare for HCC [40] and was reported only in about 2.9% of cases [41]. Despite its low incidence, it might be of relevance for HCC when considering colorectal cancer, where the percentage of MSI-H status is also observed in a minority of patients. Here pembrolizumab gained approval and is of high therapeutic relevance for this subgroup of patients.

Further potential biomarkers of response to immunotherapy such as tumor mutational burden [42], the detection of a T-cell-inflamed gene expression profile [43], or alterations of β-catenin status [44] are being explored as potential biomarkers of response to immune treatment in HCC. Transforming growth factor-β has been recently reported as a determinant of response to PD-L1 blockage [45, 46], also setting a rationale to establish therapies that block both transforming growth factor-β and PD-L1 [46, 47, 48].

Until reliable molecular biomarkers of response to the action of the several different agents are established, the clinical characteristics of patients may be used to guide the choice of treatment in HCC patients. A recently published study suggests that the etiology of the underlying liver disease may predict response to immunotherapy and, in particular, that immunological treatment might be less effective in NASH-related HCCs [49].

Despite of the fact that most mutations observed in HCC are not targetable [50], we strongly recommend to screen for targetable mutations, especially in the light of encouraging results from tumor-agnostic therapeutic approaches such as larotrecinib that demonstrated impressive tumor-agnostic effects in malignancies positive for tropomyosin receptor kinase gene fusions [51].

cMET, the hepatocyte growth factor receptor, was investigated already in former studies by the use of tivantinib. This approach failed in a large clinical trial [52]. However, later on, it became clear that tivantinib was not a pure cMET inhibitor and that its cytotoxic effects were not related to cMET inhibition [53]. Therefore, further studies using more specific cMET inhibitors such as tepotinib are under investigation [46] and have already delivered first promising results in cMET-overexpressing HCCs after sorafenib treatment [54]. FGF19, a potential driver of HCC, and its inhibition by the blockage of its receptor FGFR4 by fisogatinib were investigated recently [46, 55]. Here, in an early phase I trial, response was exclusively observed in FGF19 expressing tumors, suggesting a potential new biomarker-based approach for the treatment of HCC [55].

### Treatment of Patients with Poor Liver Function

The presence of an impaired liver function (as defined by the Child-Pugh score of 7 or higher) represents an exclusion criterion in most clinical trials. This is due to the fact that an impaired liver function is associated with poor prognosis, a factor which would heavily bias the evaluation of the anticancer efficacy. In clinical practice, this leaves an important gap in the therapy of patients with Child-Pugh B stage. Real-world data analyses suggest that immunotherapy might be beneficial in patients with impaired liver function [56, 57]. In addition, a mOS of 7.6 months was reported for patients with Child-Pugh B who received nivolumab in one of the arms (cohort 5) of the CheckMate 040 trial and compares favorably versus historical mOS data from patients with CP-B treated with sorafenib (2.5–5.4 months) [58]. Furthermore, this study showed that 5 patients (4 of whom experienced an objective radiological response) showed an amelioration of liver function to Child-Pugh A, indicating that response on immunotherapy might translate to improvement of liver function in some cases [58].

One of the most remarkable developments of systemic treatment is represented by the increasing rates of objective response (and complete responses) observed in immunotherapy-based regimens. This has an obvious relevance concerning the use of immunotherapy perioperative or neoadjuvant treatment in patients with borderline-unresectable tumors. However, high response rates may be of relevance in the treatment of patients with a poor liver function, which is due to a high intrahepatic tumor load. In clinical practice, this might translate in the decision to treat patients with otherwise good performance status and high intrahepatic tumor load with the hope that response will translate into an amelioration of liver function.

### Treatment of Patients in the BCLC-B Stage

**Fig. 2:**
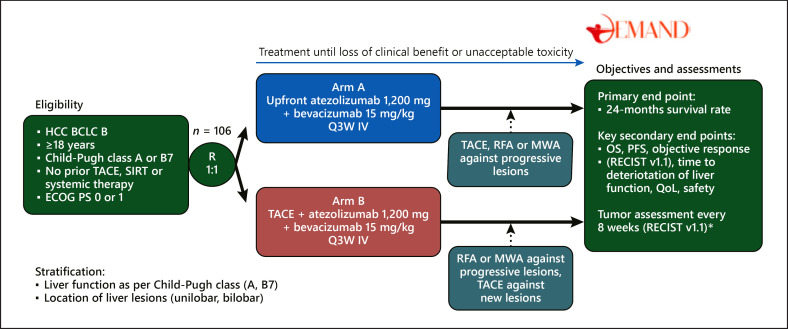
Study design of the DEMAND trial. The figure illustrates the study design of the DEMAND trial that will address efficacy of atezo/bev with or without TACE in patients with HCC in stage BCLC-B; *6 weeks after treatment initiation and every 8 weeks thereafter. BCLC, Barcelona-Clinic Liver Cancer; ECOG PS, Eastern Cooperative Oncology Group performance status; HCC, hepatocellular carcinoma; IV, intravenous; MWA, microwave ablation; N, sample size; OS, overall survival; PFS, progression-free survival; Q3W, once every 3 weeks; QoL, quality of life; R, randomization; RECIST v1.1, Response Evaluation Criteria in Solid Tumors version 1.1; RFA, radiofrequency ablation; SIRT, selective internal radiation treatment; TACE, transarterial chemoembolization. Clinical Trial Registration: NCT04224636 (ClinicalTrials.gov) *Reference: DEMAND* [60].

The increasing efficacy of systemic treatment exemplified by the long survival and high response rates under atezo/bev [17] compares favorably with the outcome of patients treated by transarterial chemoembolization (TACE) which does not exceed 20 months in real-life conditions [59]. Suboptimal patients' selection may be responsible for the limited survival of patients treated with TACE reported in this study, and the increasing effectiveness of systemic treatment raised the question of whether atezo/bev should be used in combination to or even instead of TACE in patients in stage BCLC-B. This question is the rationale for the DEMAND trial (NCT04224636) [60], a randomized study currently being conducted in Germany, investigating the efficacy of atezo/bev alone or in combination with TACE in patients with unresectable HCC (Fig. 2). In arm A, atezo/bev is initiated up-front and followed by on-demand locoregional or ablation treatment specifically directed against progressive lesions only. Patients randomized in arm B will receive TACE followed by atezo/bev. In this study design, performing locoregional treatment directed to singular lesions nonresponsive to systemic therapy should help avoiding possible collateral damage to nontumor liver parenchyma. On the other hand, the release of tumor-specific antigens caused by TACE is hypothesized to boost the effect of immunotherapy. In the phase III ABC-HCC trial (NCT04803994), patients will be randomized to receive TACE alone versus atezo/bev alone. These studies along with several other ongoing studies with other immunotherapy-based combinations [61] will provide information on the potential benefits of the extension of the use of atezo/bev to the treatment of BCLC-B patients.

### Downstaging, Bridging to Transplantation

The high response rates observed under treatment with immunotherapy is now frequently downstaging tumors to dimensions potentially allowing the use of local ablation and surgical resection, and in some cases, the patient may become eligible for transplantation [62, 63]. Although the use of checkpoint inhibitors as a bridging option prior to transplantation cannot be recommended until more data become available, some recently published reports [62, 63] indicate that immunotherapy may be used safely in the pretransplant setting. In one case [62], nivolumab was initiated after liver resection and the sequential treatment with sorafenib and regorafenib. After the patient responded to nivolumab, he was put on the waiting list for liver transplantation after a 6-week washout period and received liver transplantation altogether 15 weeks after the last cycle of nivolumab. In this case, no signs of recurrence or graft rejection were seen. Another report was published on a case series of 9 patients receiving nivolumab prior to transplantation [63]. Interestingly, in this series, 8 patients received their last cycle of nivolumab within 4 weeks before transplantation. Only one patient developed a mild rejection, which was possibly related to low tacrolimus levels. These reports indicate that checkpoint inhibitor-based systemic treatment may be used in the setting of liver transplantation in selected patients.

## Discussion and Conclusions

Systemic therapy for HCC is undergoing a rapid evolution due to the availability of several effective agents used in different lines of treatment. A peculiarity of HCC is represented by the fact that most patients suffer from concurrent liver disease and that tumor progression invariably causes a potentially fatal worsening of liver function. Therefore, identifying biomarkers capable of predicting which of the available agents will be most effective in individual patients at the earliest time point has attained importance. Here, studies need to address which sequences of therapy optimize survival of patients with HCC. Another important development of modern systemic treatment is represented by the increasing rates of objective response, especially in patients treated with immune-checkpoint inhibitors. It may become an important topic to clarify if patients with high tumor burden may also profit from reduction of tumor mass in respect of liver function. If yes, it may extend the therapeutic spectrum to more advanced disease stages for selected patients that were previously not treatable due to their liver function related to tumor burden. Before the advent of immunotherapies, systemic therapies in “high-burden” HCC patients were often not feasible due to high rates of side effects related to impaired liver function. Here, immunotherapies, as a class that show side effects more related to the predisposition of immune system rather than to liver function, raise the question whether for these former untreatable patients, an extension of therapeutic framework will become practicable. This becomes particularly relevant when complete responses are reported in 12% under therapy with atezo/bev, rates that are far above responses seen before [17].

Most clinical trials in HCC were restricted to patients with Child-Pugh A liver function. In clinical practice, this leaves an important gap in the therapy of patients with Child-Pugh B stage. Here, real-world analyses reveal practicability of immunotherapy in these patients [56, 57].

Ongoing studies like the DEMAND or ABC-HCC study will provide first insights if systemic therapies may migrate to earlier stages (BCLC-B). It will be challenging to shape the therapeutic sequence for patients that migrate from advanced stages to intermediate or even earlier stages under such effective treatments and may become candidates for resection or even liver transplantation. In our opinion, prospective studies should be conducted early to gain evidence how to treat these patients in an optimal way and whether these concepts will translate into an improvement of survival. Furthermore, it will become an interesting question if local ablative therapies could be re-evaluated when there are meaningful responses to pharmacotherapy in order to improve prognosis.

In conclusion, we face an interesting time with a much stronger armentarium of systemic therapies, and we would like to encourage the scientific community to undertake prospective studies to gain evidence on these issues discussed here in order to optimize the treatment of HCC patients in the future.

## Conflict of Interest Statement

F.P.R. has received honoraria for lectures and travel support from the Falk Foundation and Gilead. N.B.K. has received reimbursement of meeting attendance fees and travel expenses from EISAI and lecture honoraria from the Falk Foundation. G.D. has received honoraria for lectures, teaching, advisory activities, and travel support from AbbVie, Alexion, Falk Foundation, Gilead, GMP Orphan, Intercept, and Novartis. A.G. has received honoraria for lectures, teaching, advisory activities, and travel support from AbbVie, Alexion, Bayer, BMS, CSL Behring, Eisai, Gilead, Intercept, Falk Foundation, Ipsen, MSD, Merz, Novartis, Pfizer, Roche, Sanofi-Aventis, and Sequana and has received research support from Intercept and Falk Foundation (NAFLD CSG) and Novartis. E.D.T. has served as a paid consultant for AstraZeneca, Bayer, BMS, EISAI, Eli Lilly & Co, Pfizer, IPSEN, and Roche. He has received reimbursement of meeting attendance fees and travel expenses from Arqule, Astrazeneca, BMS, Bayer, Celsion, and Roche and lecture honoraria from BMS and Falk Foundation. He has received third-party funding for scientific research from Arqule, AstraZeneca, BMS, Bayer, Eli Lilly, and Roche. MODW received lecture honoraria from the Falk Foundation. All the other authors declare that they have no conflict of interest.

## Funding Sources

The authors did not receive any specific grants for this manuscript.

## Author Contributions

All the authors contributed to the literature review and search, writing, formatting, and editing of the manuscript. F.R. designed the figures, answered the questions of the peer reviewers, and replied to their comments with approval of all the authors. All the authors approved the submitted manuscript.

## Figures and Tables

**Table 1:** First-line therapies

Drug	Sorafenib	Lenvatinib	Atezolizumab/bevacizumab
Name of the study	SHARP, Asia-Pacific, REFLECT, and IMbrave150	REFLECT	IMbrave150

NCT	NCT00105443, NCT00492752, NCT01761266, and NCT03434379	NCT01761266	NCT03434379

Phase	3	3	3

Mechanism of action	Multi-kinase inhibitor targeting Raf-1, B-Raf, VEGF receptors 1–3, and PDGF receptor-β	Multi-kinase inhibitor targeting VEGF receptors 1–3, FGF receptors 1–4, PDGF receptor-α, RET, and KIT	anti-PD-L1/anti-VEGF

Dosage	800 mg/d	8 mg/d (<60 kg b.w.) and 12 mg/d (>60 kg b.w.)	Atezolizumab 1200 mg/d and bevacizumab 15 mg/kg Q3W

Patients, *n*	602 (SHARP) and 271 (Asia-Pacific)	954	501

Comparator	Placebo	Sorafenib	Sorafenib

Ratio of randomization (number)	1:1 (299:303) (SHARP) and 2:1 (150:76) (Asia-Pacific)	1:1 (478:476)	2:1 (336:165)

Child-Pugh	A (95%) and B (5%) (SHARP) and A (97%) and B (3%) (Asia-Pacific)	A (99%) B (1%)	A only (100%)

ECOG PS	≤2 (SHARP) and ≤2 (Asia-Pacific)	0 or 1	0 or 1

Etiology (viral (HBV and HCV) vs. nonviral)	Circa 50% nonviral (SHARP) and circa 20% nonviral (Asia-Pacific)	Circa 30% nonviral	30% nonviral

ORR	2% (SHARP), 3.3% (Asia-Pacific), 6.5% (REFLECT), and 11.3% (IMbrave150)	18.8%	29.8%

ORR (mRECIST)	na (SHARP), na (Asia-Pacific), 12.4% (REFLECT), and 13.9% (IMbrave150)	40.6%	35.4%

PR	2% (SHARP), 3.3% (Asia-Pacific), 6% (REFLECT), and 10.7% (IMbrave150)	18%	22.1%

PR (mRECIST)	na (SHARP), na (Asia-Pacific), 12% (REFLECT), and 11.4% (IMbrave150)	38%	23.4%

CR	0% (SHARP), 0% (Asia-Pacific), <1% (REFLECT, and; 0.6% (IMbrave150)	<1%	7.7%

CR (mRECIST)	na (SHARP), na (Asia-Pacific), 1% (REFLECT), and 2.5% (IMbrave150)	2%	12.0%

SD	71% (SHARP), 54% (Asia-Pacific), 53% (REFLECT), and 43.4% (IMbrave150)	54%	44.2%

SD (mRECIST)	na (SHARP), na (Asia-Pacific), 46% (REFLECT), and 41.1% (IMbrave150)	33%	37.2%

DOR	na (SHARP), na (Asia-Pacific) na (REFLECT), and 14.9 mo (IMbrave150)	na	18.1 mo

DOR (mRECIST)	na (SHARP), na (Asia-Pacific), na (REFLECT), and 12.6 mo (IMbrave150)	na	16.3 mo

mOS	10.7 mo (SHARP), 6.5 mo (Asia-Pacific), 12.3 mo (REFLECT), and 13.4 mo (IMbrave150)	13.6 mo	19.2 mo

Relevant aspects of inclusion or exclusion criteria	Life expectancy of 12 weeks or more^i^; adequate hematologic function (platelet count, ≥60 × 10^9^ per liter; hemoglobin, ≥8.5 g per deciliter; prothrombin time international normalized ratio, ≤2.3)^i^; adequate hepatic function (albumin), ≥2.8 g per deciliter^i^; total bilirubin ≤3 mg per deciliter^i^; ALT and AST, ≤5 times the upper limit of the normal range^i^; and adequate renal function (serum creatinine, ≤1.5 times the upper limit of the normal range)^i^	Controlled blood pressure (≤150/90 mm Hg)^i^; adequate liver function (albumin ≥2.8 g/dL, bilirubin ≤3.0 mg/ dL, AST, ALT and alkaline phosphatase ≤5 times the upper limit of normal)^i^; adequate bone marrow (hemoglobin ≥8.5 g/dL, platelet count ≥75 × 10^9^ per L; absolute neutrophil count ≥1.5 × 109 per L)^i^; blood (international normalized ratio ≤2.3); and renal and pancreatic function. Patients with 50% or higher liver occupation, obvious invasion of the bile duct, or invasion at the main portal vein were excluded from the study^e^. No systemic treatment for HCC in history^i^	History of autoimmune disease^e^; coinfection with hepatitis B or hepatitis C virus^e^; untreated or incompletely treated esophageal; or gastric varices (assessed with esoph-agogastroduodenoscopy and treated according to local clinical practice) with bleeding or high risk of bleeding^e^

Approved by FDA	Yes	Yes	Yes

Approved by EMA	Yes	Yes	Yes

The table illustrates the results of the main first-line trials in HCC. The table gives an overview about relevant endpoints of the trials mentioned here. We would like to highlight at this point that results are not directly comparable as most of them were generated from different studies. Sorafenib served as comparator in most trials. Therefore, we included the response and efficacy data from different trials in this table for sorafenib (SHARP; Asia-Pacific, REFLECT, and IMbrave150). Results from an update of the IMbrave150 study presented as a conference article are reported in this table. Inclusion criteria are marked with

i and exclusion criteria are marked with

e . b.w., body weight; CR, complete response; DOR, duration of response; EMA, European Medicines Agency; FDA, Food and Drug Administration; HBV, hepatitis B virus; HCC, hepatocellular carcinoma; HCV, hepatitis C virus; ECOG PS, Eastern Cooperative Oncology Group performance status; mo, months; mOS, median overall survival; mRECIST, modified Response Evaluation Criteria in Solid Tumors; na, not available; NCT, National Clinical Trial number; ORR, overall response rate; PR, partial response; Q3W, every 3 weeks; SD, stable disease. References: *SHARP Trial* [12]; *Asia-pacific* [13]; *REFLECT Trial* [14]; *IMbrave 150 Trial* [5, 11].

**Table 2:** Approved second-line therapies

Drug	Regorafenib	Cabozantinib	Ramucirumab
Name of the study	RESORCE	CELESTIAL	REACH-2

NCT	NCT01774344	NCT01908426	NCT02435433

Phase	3	3	3

Mechanism of action	Multi-kinase inhibitor targeting Raf-1, B-Raf, VEGF receptors 1–3, FGFR, Kit, ret, and PDGF receptor-β	Multi-kinase inhibitor targeting VEGF receptors 1–3, MET, and AXL	Recombinant IgG1 monoclonal antibody against VEGFR-2

Dosage	160 mg/d (3 weeks on/1 week off)	60 mg/d	8 mg/kg Q2W

Patients, *n*	573	707	292

Comparator	Placebo	Placebo	Placebo

Ratio of randomization (number)	2:1 (379:194)	2:1 (470:237)	2:1 (197:95)

Child-Pugh	A (98%) B (1%)	A (98%) B (1%)	A (only)

ECOG PS	0 or 1	0 or 1	0 or 1

Etiology (viral [HBV and HCV] vs. nonviral)	Circa 40% nonviral	Circa 40% nonviral	Circa 40% nonviral

ORR	7%	4%	4.6%

ORR (mRECIST)	11%	na	na

PR	7%	4%	4.6%

PR (mRECIST)	10%	na	na

CR	0%	0%	0%

CR (mRECIST)	1%	na	na

SD	59%	60%	59.9%

SD (mRECIST)	54%	na	na

DOR	na	na	na

DOR (mRECIST)	3.5 mo	n.a	n.a

mOS	10.6 mo	10.2 mo	8.5 months

Relevant aspects of inclusion or exclusion criteria	Adults with HCC who tolerated sorafenib (≥400 mg/day for ≥20 of last 28 days of treatment)^i^; progression on sorafenib^i^; AND Child-Pugh A liver function ^i^	Received sorafenib ^i^; progression on at least prior systemic treatment for HCC^i^; adequate hematologic and renal function; total bilirubin ≤2 mg/dU^i^; serum albumin ≥2.8 g/dL^i^; ALT and AST <5.0 ULN^i^; HbA1c ≤8%^i^, fasting serum glucose ≤160 mg/dU; more than 2 prior systemic therapies^e^; concomitant anticoagulation^e^; and QTcF >500ms^e^	1 Received sorafenib^i^; AFP concentrations of 400 ng/mL or greater^i^

Approved by FDA	Yes	Yes	Yes

Approved by EMA	Yes	Yes	Yes

The table illustrates the results of the second-line trials that led to FDA and EMA approval. Inclusion criteria are marked with

i and exclusion criteria are marked with

e . ALT, alanine aminotransferase; AST, aspartate aminotransferase; CR, complete response; DOR, duration of response; EMA, European Medicines Agency; FDA, Food and Drug Administration; HbA1c, glycated hemoglobin; HBV, hepatitis B virus; HCC, hepatocellular carcinoma; HCV, hepatitis C virus; ECOG PS, Eastern Cooperative Oncology Group performance status; mo, months; mOS, median overall survival; mRECIST, modified Response Evaluation Criteria in Solid Tumors; na, not available; NCT, National Clinical Trial; ORR, overall response rate; PR, partial response; Q2W, every 2 weeks; QTcF, corrected QT interval; SD, stable disease; VEGF, vascular endothelial growth factor; AFP, α-fetoprotein. References: *RESORCE* [6]; *CELESTIAL* [7]; *REACH-2* [8].

**Table 3:** Second-line therapies not approved by EMA

Drug	Nivolumab	Pembrolizumab	Nivolumab and ipilimumab
Name of the study	CheckMate 040	Keynote-240	CheckMate 040

NCT	NCT01658878	NCT02702401	NCT01658878

Phase	1/2	3	1/2

Mechanism of action	Monoclonal antibody targeting PD-1	Monoclonal antibody targeting PD-1	Monoclonal antibodies targeting PD-1 and CTLA-4

Dosage	3 mg/kg Q2W	200 mg Q3W	Nivolumab 1 mg/kg plus ipilimumab 3 mg/kg Q3W (4 doses) followed by nivolumab 240 mg Q2W (arm A)

Number of patients	214 (57 after sorafenib)	413	148

Comparator	No comparator	Placebo	Two different dosages of nivolumab and ipilimumab

Ratio of randomization (number)	No comparator	2:1 (278:135)	1:1:1

Child-Pugh	A (98%) B (2%)	A (99%) B (<1%)	A (only)^$^

ECOG PS	0 or 1	0 or 1	0 or 1

Etiology (viral [HBV and HCV] vs. nonviral)	Circa 50% nonviral	Circa 60% nonviral	Circa 30% nonviral^$^

ORR	21%^§^	18.3%	32%^$^

ORR (mRECIST)	na	na	na

PR	18%^§^	16.2	24%^$^

PR (mRECIST)	na	na	na

CR	4%^§^	2.2	8%^$^

CR (mRECIST)	na	na	na

SD	40%^§^	43.9	18%^$^

SD (mRECIST)	na	na	na

DOR	Not reached	13.8 mo	17.5 mo

DOR (mRECIST)	na	na	na

mOS	13.2 mo	13.9 mo	22.8 mo

Relevant aspects of inclusion or exclusion criteria	Patients with HBV infection were required to be receiving effective antiviral therapy and have a viral load less than 100 IU/mL^i^; patients who had previously been treated with an agent targeting T-cell co-stimulation or checkpoint pathways (including those targeting PD-1, PD-L1 or PD-L2, CD137, or CTLA-4) were excluded^e^	Patients who had received prior immunotherapy, including anti-PD-1, anti-PD-L1, or anti-PD-L2 agents or previous systemic therapy for HCC in the advanced setting other than sorafenib were excluded^e^; patients with clinically apparent ascites on physical examination, main portal vein invasion or inferior vena cava or cardiac involvement of HCC on the basis of imaging, or clinically diagnosed hepatic encephalopathy within the past 6 months were excluded^e^	Patients who had active coinfection with HBV and HCV, or HBV and HDV were not eligible^e^

Approved by FDA	Approval withdrawn [30]	Yes	Yes

Approved by EMA	No	No	No

	^§^results from a subgroup (*N* = 57) that were progressors on sorafenib and indicate a second-line setting		^$^Results from arm A (*n* = 50) are reported here

The table illustrates the results of the second-line trials that led to approval by the FDA but currently not by the EMA. Inclusion criteria are marked with

i and exclusion criteria are marked with

e . CR, complete response; DOR, duration of response; EMA, European Medicines Agency; FDA, Food and Drug Administration; HBV, hepatitis B virus; HCC, hepatocellular carcinoma; HCV, hepatitis C virus; ECOG PS, Eastern Cooperative Oncology Group performance status; mo, months; mOS, median overall survival; mRECIST, modified Response Evaluation Criteria in Solid Tumors; na, not available; NCT, National Clinical Trial number; ORR, overall response rate; PR, partial response; Q2W, every 2 weeks; Q3W, every 3 weeks; SD, stable disease; PD-L1, PD-1 ligand; CTLA-4, cytotoxic T-lymphocyte antigen. References: *CheckMate 040* [20]; *Keynote-240* [22]; *CheckMate 040* [23].
